# Cooperation or Conflict? Molecular and Physiological Cross-Talk Between the Aryl Hydrocarbon and Vitamin D Receptors

**DOI:** 10.3390/ph19091416

**Published:** 2026-09-08

**Authors:** Mohammed A. Alqahtani

**Affiliations:** Department of Pharmacology, College of Pharmacy, King Khalid University, P.O. Box 960, Abha 61421, Saudi Arabia; mualalqahtani@kku.edu.sa

**Keywords:** vitamin D, calcitriol, aryl hydrocarbon receptor, VDR, ARNT, nuclear receptor, xenobiotic signaling, immunomodulation, CYP enzymes, p53, cross-talk

## Abstract

The aryl hydrocarbon receptor (AHR) and the vitamin D receptor (VDR) were long regarded as independent transcription factors governing distinct physiology—xenobiotic sensing and calcium–vitamin D homeostasis, respectively. AHR, a basic helix–loop–helix/PAS protein, heterodimerizes with ARNT and binds xenobiotic response elements (XREs) to drive cytochrome P450 genes such as *CYP1A1*; VDR, a nuclear receptor activated by 1,25-dihydroxyvitamin D_3_, heterodimerizes with RXR and binds vitamin D response elements (VDREs). Although their genes reside on separate chromosomes (*AHR*, Chr 7; *VDR*, Chr 12), an integrated view recognizes the two pathways as extensively cross-regulatory. This review synthesizes the molecular, immunological, and tissue-level evidence for VDR–AHR interplay. At the molecular level, the receptors cooperate at composite promoter architectures—most notably an everted-repeat VDRE positioned adjacent to an XRE in the *CYP1A1* promoter—while AHR ligands reciprocally enhance *CYP24A1*-mediated catabolism of active vitamin D. Tryptophan metabolism provides a bidirectional hub: kynurenine and the UVB photoproduct FICZ serve as endogenous AHR ligands whose balance, modulated by VDR, shapes signaling output. The tumor suppressor p53 functions as a shared upstream regulator coupling genotoxic stress to both receptors, with convergence on the *CDKN1A* (p21) checkpoint. Functionally, AHR and VDR converge on the regulatory T cell (Treg)/Th17 axis to influence immune tolerance: sustained AHR activation by TCDD favors Foxp3^+^ Treg differentiation, transient FICZ-driven activation promotes Th17 responses, and VDR reinforces the tolerogenic arm while independently repressing IL-17. The receptors further cooperate in maintaining intestinal epithelial barrier integrity and NF-κB restraint, with parallel impairment in inflammatory bowel disease, and are co-activated in skin by solar UVB, which simultaneously generates vitamin D_3_ and the AHR ligand FICZ within keratinocytes. In cancer, VDR acts as a tumor suppressor, AHR exhibits context-dependent pro- and anti-tumor roles, and a three-way AHR–VDR–p53 interaction—inverted by mutant p53—forms a critical regulatory node. Throughout, the direction and magnitude of cross-talk prove highly dependent on cell type, ligand identity and kinetics, and species—distinctions often underappreciated in the literature. Clarifying these context-specific determinants is essential for translating AHR–VDR cross-regulation into rational therapies in autoimmunity, mucosal inflammation, dermatology, and oncology.

## 1. Introduction

The aryl hydrocarbon receptor (AHR) belongs to the basic helix–loop–helix (bHLH)/Per-ARNT-Sim (PAS) protein family [1]. Historically defined as a sensor for halogenated and polycyclic aromatic hydrocarbons—including the prototypical ligand TCDD—the AHR responds to a diverse repertoire of endogenous and dietary ligands such as tryptophan metabolites and microbiota-generated indoles. Upon ligand binding in the cytoplasm, AHR translocates to the nucleus and heterodimerizes with ARNT, binding dioxin/xenobiotic response elements (XREs) to activate target genes including cytochrome *P450 1A1, 1A2* and *1B1* (*CYP1A1, CYP1A2*, and *CYP1B1*) [2,3,4,5].

Vitamin D has long been recognized as a pleiotropic hormone with actions that extend far beyond its canonical role in calcium homeostasis and bone mineralization [6,7]. The biologically active form, 1α,25-dihydroxyvitamin D3 (1,25D or calcitriol), signals through the vitamin D receptor (VDR), a member of the nuclear receptor (NR) superfamily. Upon ligand binding, VDR heterodimerizes with the retinoid X receptor (RXR), and the complex binds vitamin D response elements (VDREs) in target gene regulatory regions, engaging co-activator complexes to modulate transcription [8,9]. VDR is expressed in virtually every nucleated cell type, underscoring the broad influence of 1,25D on gene regulation across tissues.

The notion that AHR and VDR operate in isolation has given way to a more integrated view. At the genomic level, analysis of the human reference genome (GRCh38) reveals that *AHR and VDR* reside on two distinct chromosomes [10,11]. This review provides a comprehensive, mechanistically grounded account of the cross-regulatory relationship between the AHR and VDR signaling axes, encompassing molecular mechanisms, physiological contexts, and translational opportunities.

## 2. Molecular Architecture of AHR and VDR Signaling

### 2.1. Chromosomal Gene Proximity and Genomic Organization

Analysis of the human reference genome (GRCh38) reveals that the target genes for AHR and VDR are located on distinct chromosomes—*AHR* on 7p21.1 (47,495 bp) and *VDR* on 12q13.11 (63,457 bp) [10,11]—confirming genetic independence at the linear DNA level. While the *VDR* and *AHR* genes are far apart in the linear sequence of DNA, it is important to consider their three-dimensional (3D) location within the cell nucleus. In the nucleus, chromosomes are not straight lines; they occupy specific, non-overlapping territories. Although the two genes, located on different chromosomes (Chr 12 and Chr 7), are genetically independent, their signaling pathways interact in a complex, context-dependent manner [10,11,12,13].

This functional relationship provides the theoretical basis for an interaction: it is possible for genes on different chromosomes to come into physical proximity in 3D space if their protein products interact, if they are co-regulated, or if they are targeted to the same active nuclear region [14]. In support of the co-regulation mechanism, electrophoretic mobility shift assay (EMSA) and chromatin immunoprecipitation (ChIP)—used to confirm physical protein–DNA binding in vitro and within intact chromatin in vivo, respectively—demonstrated that VDR directly occupies an everted repeat 8 (ER8) motif within the *CYP1A1* promoter, a canonical *AHR* target locus. Combined activation of *AHR* and *VDR* produced cooperative transcriptional outcomes at this shared site that neither receptor drives alone, an effect abolished by *VDR* knockdown, VDR antagonism, and AHR antagonism [15]. However, whether this functional co-regulation at shared target loci translates into physical proximity of the *VDR* and *AHR* gene loci themselves within nuclear space remains to be demonstrated and represents an important open question in the field.

### 2.2. The Aryl Hydrocarbon Receptor Pathway

AHR is a bHLH/PAS transcription factor. In its unliganded state, cytoplasmic AHR is maintained in a high-affinity conformation by a chaperone complex comprising two HSP90 molecules, p23, and the immunophilin-like protein XAP2. Ligand binding induces a conformational change, dissociating the chaperone complex and enabling nuclear import. In the nucleus, AHR heterodimerizes with ARNT, and the AHR–ARNT heterodimer binds the XRE core sequence 5′-GCGTG-3′ to activate target genes. Chromatin remodeling is achieved through recruitment of p300/CBP, SRC-1, and the Mediator complex. The AHR repressor protein (AHRR) provides negative feedback by competing with AHR for ARNT dimerization [2,16]. Recent crystal structures of the AHR–ARNT–DNA complex bound to six distinct agonists—including tapinarof, FICZ, benzo[a]pyrene, β-naphthoflavone, indigo, and indirubin—have resolved the structural basis of AHR’s ligand promiscuity, revealing eight conserved PAS-B pocket residues that undergo dynamic rearrangements to accommodate structurally diverse ligands via hydrophobic and π–π interactions, and identifying a segment of AHR that transitions from chaperone engagement to ARNT heterodimer stabilization upon activation [17]).

The molecular basis of AHR cytoplasmic stability has been the subject of an active and evolving literature. In its inactive state, AHR is held in a cytoplasmic complex with two molecules of Hsp90, the AHR-interacting protein XAP2, and the co-chaperone p23 [16]. The classical view, developed initially in yeast and in vitro systems, held that p23 functions principally as an Hsp90 co-chaperone, inhibiting Hsp90 ATPase activity and thereby stabilising the ATP-bound Hsp90–AHR client complex without directly contacting the receptor [16,18]. More recent biochemical and cellular work has revised this view. Knockdown of p23 in mouse Hepa1c1c7, human Hep3B, and human HeLa cells reduces AHR protein levels through a non-classical, Hsp90-independent degradation pathway that is not blocked by MG132, indicating that p23 directly stabilises AHR by a mechanism distinct from canonical proteasomal turnover [19]. Subsequent mapping by GST–p23 fusion pull-downs and AHR truncation constructs localised the direct p23–AHR interaction to AHR amino acids 1–216 and the N-terminal 110 amino acids of p23; Hsp90-binding-deficient p23 mutants still rescued AHR levels, and Hsp90 knockdown to ~55% of normal did not appreciably alter AHR protein abundance, indicating that p23—not Hsp90—is the primary chaperone determinant of AHR protein stability in human cells [20]. The in vivo relevance of this cell-line evidence remains debated, however: p23-null mouse embryos display stable hepatic AHR protein levels and normal β-naphthoflavone- and TCDD-induced *Cyp1a1* and *Cyp1a2* mRNA expression, suggesting that the requirement for p23 may be cell-type- and context-dependent [21]. The precise step at which p53 intersects this chaperone-controlled stability—whether through translation enhancement, modulation of p23–AHR binding, or alteration of ubiquitination pathways—remains to be determined by direct half-life measurements.

Endogenous AHR ligands of physiological importance include tryptophan catabolites (kynurenine, kynurenic acid, FICZ), arachidonic acid metabolites, and bilirubin. The tryptophan photoproduct FICZ, generated in skin upon UVB irradiation, is a particularly potent endogenous agonist with strong Th17-promoting activity. TCDD and related polyhalogenated compounds serve as the prototypical exogenous AHR ligands used extensively in mechanistic studies [2,16,22].

### 2.3. The Vitamin D Receptor Pathway

VDR is a 427-amino acid protein with a modular architecture common to nuclear receptors: an N-terminal A/B domain, a DNA-binding domain (DBD) containing two zinc fingers, a hinge region, and a C-terminal ligand-binding domain (LBD). The LBD harbors the ligand-dependent activation function-2 (AF-2) helix, whose conformational repositioning upon 1,25D binding creates a surface for co-activator recruitment. Structural studies at 1.8 Å resolution reveal that 1,25D fits into a hydrophobic pocket within the LBD, making van der Waals contacts with hydrophobic residues lining the binding cavity [23].

The VDR–RXR heterodimer preferentially binds VDREs consisting of two hexanucleotide half-sites arranged as direct repeats spaced by three nucleotides (DR3 motif) [24]. Co-activator complexes recruited by activated VDR–RXR include SRC-1 [25], SRC-2 and SRC-3 [26], DRIP205/MED1 [27], and the acetyltransferases CBP/p300 [28]. Transcriptional repression is mediated through interactions with NCoR [29] and SMRT [30]. The major 1,25D-catabolizing enzyme, CYP24A1, is itself a direct VDR target gene, creating a negative feedback loop that limits signaling duration [7,31,32].

### 2.4. The Shared Co-Factor Network: A Molecular Nexus

A critical convergence point of AHR and VDR signaling is their shared reliance on transcriptional co-activators. Both receptors recruit members of the SRC/p160 family for full transcriptional activity at their respective target gene promoters. For AHR, SRC-1 was the first coactivator identified, shown to interact with the Q-rich subdomain of AHR and enhance TCDD-dependent transcription [33], with subsequent ChIP-based work demonstrating broader recruitment of SRC-1, SRC-2 (NCoA-2), and CBP by the AHR/ARNT complex at endogenous target gene promoters [34]. For VDR, ChIP-based studies in intact osteoblasts demonstrated ligand-dependent, cyclic recruitment of SRC-1, SRC-2, SRC-3, CBP, and p300 to VDR target gene promoters, accompanied by histone H4 acetylation [28]. The overlap in coactivator dependency—particularly SRC-1, SRC-2, and CBP—raises the possibility that when both pathways are simultaneously activated in the same cell by their respective ligands, an AHR ligand such as benzo[a]pyrene and the VDR ligand 1,25(OH)_2_D_3_, competition for these limited co-activator pools could constitute a mechanism of cross-regulatory interference. No study has yet directly demonstrated this competition between AHR and VDR; however, the phenomenon of coactivator squelching is well established for other simultaneously activated nuclear receptors, where activation of one receptor depletes the available pool of shared p160 coactivators and attenuates the transcriptional output of the other [35,36]. Evidence that VDR itself can exert coactivator competition has been demonstrated in hepatocytes, where ligand-activated VDR is associated with decreased occupancy of PGC-1α and GRIP-1 at the *CYP7A1* promoter chromatin—directly measured by ChIP assay in primary human hepatocytes—coinciding with increased recruitment of corepressors NCoR-1 and SMRT, consistent with VDR-mediated displacement of HNF4α-associated coactivators to suppress *CYP7A1* transcription [37]. Mammalian two-hybrid assays—which detect protein–protein interactions in living cells via reporter gene output—further showed that SRC-1, alongside PGC-1α, participates in the VDR–HNF4α regulatory complex in a ligand-dependent manner, and that corepressor recruitment by NCoR-1 and SMRT disrupts this SRC-1/PGC-1α–HNF4α complex upon VDR activation, consistent with a broader model of coactivator competition at this locus [38].

## 3. Molecular Mechanisms of Cross-Regulatory Signaling

### 3.1. AHR-VDR Cross-Regulatory Modulation on CYP1A1 and CYP24A1

CYP1A1 is the most extensively studied transcriptional target of the AHR signalling pathway and is widely regarded as the canonical biomarker of AHR activation. Upon ligand binding—whether by hazardous environmental chemicals such as TCDD [39] and benzo[a]pyrene [40], or by endogenous ligands such as FICZ [41] and kynurenine [41], AHR dissociates from its cytoplasmic chaperone complex, translocates to the nucleus, heterodimerises with ARNT, and binds xenobiotic response elements (XREs) in the CYP1A1 enhancer region, driving robust and rapid transcriptional induction [2,16]. This response is so consistent across cell types, species, and experimental systems that *CYP1A1* mRNA induction and its associated enzymatic activity have become the gold standard functional readouts for AHR pathway activation in both basic research and regulatory toxicology. The sensitivity, dynamic range, and reproducibility of CYP1A1 induction have made it an indispensable tool for characterising AHR ligands, quantifying toxic equivalency factors (TEFs) of dioxin-like compounds, and detecting environmental contamination [42]. Its role as a universal AHR marker, however, does not imply a uniform regulatory outcome across all cellular and signalling contexts, as illustrated by its complex cross-regulation by the vitamin D receptor pathway.

Rather than a simple suppressive relationship, the cross-talk between AHR and VDR signalling at the *CYP1A1* locus is cooperative and promoter-architecture dependent. Studies in human monocyte/macrophage-derived cells (U937 and THP-1) demonstrate that 1,25D enhances rather than suppresses AHR-driven *CYP1A1* transcription [15]. The co-treatment with 1,25(OH)_2_D_3_ and the AHR agonist benzo[a]pyrene increased *CYP1A1* mRNA, protein levels, and enzymatic activity more effectively than AHR activation alone, and this combined induction was abrogated by VDR knockdown, VDR antagonists, and the AHR antagonist α-naphthoflavone, confirming that both receptors are required [15]. The mechanistic basis for this cooperativity lies within the *CYP1A1* promoter, where an everted repeat (ER8-type) Vitamin D response element at −506 to −525 bp sits immediately adjacent to a canonical XRE (−489 to −495 bp). EMSA and ChIP assays confirmed that the VDR/RXR complex binds directly to this ER8 motif in a ligand-dependent manner [15]. The proximity of these two response elements allows VDR and AHR/ARNT complexes to interact cooperatively at the same promoter region, producing synergistic transcriptional activation rather than competition [15].

A further layer of this bidirectional cross-talk was demonstrated by the same group. AHR activation by benzo[a]pyrene also enhances 1,25D-dependent induction of CYP24A1, the principal enzyme of vitamin D catabolism [43], in THP-1 and MCF-7 breast cancer cells, and this effect was also observed with TCDD as the AHR agonist [13]. This creates a reciprocal relationship in which AHR ligand exposure accelerates the degradation of 1,25D while simultaneously amplifying its effect on CYP1A1.

Taken together, these findings demonstrate that AHR and VDR do not function as simple mutual modulators. Instead, their co-activation results in enhanced expression of this xenobiotic-metabolising enzyme, with important implications for the carcinogenic risk associated with simultaneous exposure to polycyclic aromatic hydrocarbons and vitamin D. Whether this cooperative relationship extends to TCDD-specific AHR activation in breast cancer cells, hepatocytes, and keratinocytes under co-treatment conditions remains to be formally established, representing an important gap in the current literature.

### 3.2. Tryptophan Metabolism as a Bidirectional Regulatory Hub

Tryptophan catabolism provides a direct bidirectional regulatory link between the AHR and VDR signalling pathways, operating through two distinct but interconnected biochemical branches: the kynurenine pathway and the ultraviolet B (UVB)-dependent photoproduct pathway. Together, these branches position the skin and the immune microenvironment as critical sites where endogenous ligand production for both receptors is simultaneously regulated by a common environmental stimulus [44,45,46,47].

#### 3.2.1. The Kynurenine Pathway

The kynurenine pathway is initiated by two rate-limiting enzymes: indoleamine 2,3-dioxygenase 1 (IDO1) and tryptophan 2,3-dioxygenase 2 (TDO2), both of which catalyse the first step in tryptophan catabolism, which is rapidly hydrolysed to kynurenine [48,49]. Kynurenine and several of its downstream metabolites, including kynurenic acid and xanthurenic acid, are biologically active endogenous AHR ligands capable of driving AHR nuclear translocation, XRE-dependent transcription, and *CYP1A1* induction in both immune and epithelial cells [48,50]. The foundational evidence for kynurenine as a direct endogenous AHR ligand was established by Opitz et al. in human glioma cells, where TDO2-derived kynurenine was shown to activate the AHR in an autocrine/paracrine fashion, suppressing anti-tumour immunity [50].

The immunological relevance of this pathway is particularly well established in dendritic cells. AHR itself is required for IDO expression in murine dendritic cells; in bone marrow-derived dendritic cells (BMDCs) from AHR-deficient mice, IDO induction was markedly impaired in response to lipopolysaccharide (LPS) and CpG stimulation, skewing naïve T-cell differentiation away from a regulatory T cell (Treg) phenotype [51]. Kynurenine, the IDO-generated tryptophan metabolite, is itself an AHR ligand that activates the receptor through dioxin response elements and promotes Treg generation in murine T cells [41], establishing a self-reinforcing loop in which AHR activation amplifies its own tolerogenic ligand supply through IDO upregulation. It is important to note, however, that AHR does not uniformly promote tolerance. The immune outcome of AHR activation is critically ligand-dependent: in murine models, TCDD drives the development of Forkhead box P3-expressing regulatory T cells (Foxp3+ Tregs) and suppresses experimental autoimmune encephalomyelitis (EAE), whereas the tryptophan-derived photoproduct FICZ instead promotes Th17 differentiation, increases IL-17A/F and IL-22 production, and accelerates onset and severity of EAE [52,53].

A proposed cross-regulatory mechanism linking VDR and AHR signalling involves the upregulation of IDO1 by 1,25D in dendritic cells, which would be expected to increase the endogenous production of kynurenine and thereby potentiate AHR-mediated immunosuppression. However, the experimental evidence for this cascade is species-dependent and currently conflicting. In a rodent model, vitamin D3 has been shown to induce IDO-expressing tolerogenic dendritic cells. Farias et al. demonstrated that bone marrow-derived dendritic cells from Lewis rats cultured with vitamin D3 acquired a tolerogenic phenotype with high IDO, IL-10, and TNFα expression and reduced MHC-II and CD80. Prophylactic adoptive transfer of these IDO^+^ DCs one day before EAE induction significantly increased the percentage of CD4^+^Foxp3^+^ regulatory T cells in lymph nodes at day 12 post-immunization, during the exacerbation phase of disease. The functional dependence of vitamin D3′s anti-EAE effect on IDO was confirmed using the competitive IDO inhibitor 1-methyl-tryptophan, which also reversed the IL-10 and TNFα phenotype of vitamin D3-treated DCs in vitro [54].

In contrast, studies in human dendritic cells have yielded the opposite finding. Pedersen et al. reported that 1,25(OH)_2_D_3_ treatment down-modulated maturation-induced IDO activity in human monocyte-derived dendritic cells from five independent donors, measured by the kynurenine-to-tryptophan ratio in culture supernatants, and concluded that high IDO activity per se cannot define the regulatory function of VD3-treated DCs [55]. This finding has been corroborated by Hafkamp et al., who demonstrated that VD3 priming of human DCs reduces IDO expression while simultaneously enhancing Treg development; selective IDO inhibition with epacadostat did not impair Treg induction, indicating that the tolerogenic effect of 1,25D in human DCs operates through IDO-independent mechanisms [56]. A mechanistic explanation for this species divergence has been proposed: IDO induction in human dendritic cells requires non-canonical NF-κB signalling [57], which 1,25D suppresses through direct transcriptional repression of RelB, demonstrated in a DC-derived cell line and murine bone marrow-derived dendritic cells [58].

Despite this controversy at the level of IDO induction in human DCs, the downstream components of the proposed cascade are individually well-established. IDO1, IDO2, and TDO catalyse the rate-limiting conversion of tryptophan to kynurenine [49], and kynurenine has been definitively identified as an endogenous AHR ligand capable of driving AHR nuclear translocation and CYP1A1 induction in tumour cells [50] and immune cells [41]. Kynurenine-driven AHR activation in turn promotes the differentiation of naïve CD4^+^ T cells into Foxp3^+^ regulatory T cells in an AHR-dependent manner, as demonstrated by Mezrich et al. using naïve T cells isolated from *AhR*-knockout mice cultured in vitro with TGF-β and kynurenine [41]. 1,25D–VDR signalling reinforces this immunological balance through an AhR-independent route. VDR directly induces Foxp3 transcription via a vitamin D response element in the mouse Foxp3 promoter, and separately suppresses Th17 IL-17A production through VDR-mediated sequestration of Runx1 [59].

Taken together, VDR and AHR are best understood not as a single linear cascade but as parallel pathways converging on Treg induction. The 1,25D → IDO1 → kynurenine → AHR bridge appears largely species-restricted to rodents, a distinction insufficiently acknowledged in the broader literature. AHR-driven tolerance is itself ligand-restricted and species-dependent: in murine cells, kynurenine and TCDD promote Foxp3^+^ Treg differentiation, whereas FICZ drives pro-inflammatory Th17 responses; in human cells, direct primary-cell evidence supports the AHR → Foxp3^+^ Treg arm only for TCDD, and only in the presence of TGF-β1 co-stimulation [60]. Any meaningful interpretation of VDR–AHR cross-talk must therefore account for both species- and ligand-specific context.

#### 3.2.2. The UVB Photoproduct Branch: FICZ and Simultaneous AHR–VDR Co-Activation

The second branch of tryptophan-derived AHR ligand production operates through direct ultraviolet photochemistry. The landmark study by Fritsche et al. established that UVB irradiation of human HaCaT keratinocytes generates the tryptophan photoproduct FICZ intracellularly. Tryptophan starvation prior to UVB exposure significantly reduced UVB-induced *CYP1A1* mRNA expression, which was restored by tryptophan re-supplementation—directly demonstrating that tryptophan acts as a UVB chromophore whose photolytic products mediate AHR-dependent signalling in skin cells [61]. UVB exposure of human skin in vivo also induces CYP1A1 at both the mRNA and protein level in epidermal keratinocytes, consistent with this mechanism operating physiologically [62]. FICZ binds the AHR ligand-binding domain with an affinity comparable to that of TCDD, a known AHR inducer (Kd ≈ 0.44 nM vs. 0.48 nM, respectively [63,64] and drives rapid AHR nuclear translocation and robust *CYP1A1* transcriptional induction [61]. The AHR-dependence of these responses was confirmed in *AHR*-knockout mouse skin, where UVB-induced *CYP1A1* induction was compromised, and by 3′-methoxy-4′-nitroflavone-mediated AHR antagonism in human HaCaT cells [61].

Importantly, FICZ is itself a substrate of CYP1A1, creating a self-limiting negative feedback loop: AHR activation by FICZ induces CYP1A1, which rapidly metabolises FICZ, thereby terminating the AHR signal and ensuring that UVB-driven AHR activation is transient [65,66]. This kinetic self-regulation fundamentally distinguishes UVB-generated FICZ from the persistent, non-metabolisable AHR ligand TCDD, and has important consequences for the magnitude and duration of *AHR* target gene induction in the skin.

#### 3.2.3. Simultaneous Activation of AHR and VDR by Solar UVB: The Skin as a Dual-Pathway Hub

The critical point of convergence is that solar UVB radiation simultaneously activates both the AHR and VDR signalling systems through entirely distinct photochemical mechanisms operating in the same tissue. The AHR pathway is activated via FICZ photogeneration, as described above [61]. In parallel, UVB photolytically converts 7-dehydrocholesterol in keratinocyte plasma membranes to pre-vitamin D_3_, which undergoes thermal isomerisation to vitamin D_3_ (cholecalciferol). Vitamin D_3_ is then sequentially 25-hydroxylated (by CYP2R1 and CYP27A1) and 1α-hydroxylated by CYP27B1; keratinocytes uniquely express CYP27B1 at higher levels than any other cell type, enabling local generation of the biologically active 1α,25-dihydroxyvitamin D_3_ (1,25(OH)_2_D_3_), which then engages the VDR [67,68].

Differential AHR and VDR target gene regulation by UVB and 1,25(OH)_2_D_3_ has been directly examined in HaCaT keratinocytes and malignant SCL-1 squamous cell carcinoma cells. UVB induced *CYP1A1* mRNA (an AHR target gene) in both cell lines, whereas *CYP24A1* mRNA (a VDR target gene) was induced by 1,25(OH)_2_D_3_ but not by UVB alone [12]. Co-treatment with 1,25(OH)_2_D_3_ and UVB further enhanced 1,25(OH)_2_D_3_-induced *CYP24A1* upregulation exclusively in malignant SCL-1 cells; this enhancement was absent in precancerous HaCaT cells, pointing to stage-dependent differences in the functional coupling of the AHR and VDR pathways during photocarcinogenesis. The AHR and VDR dependence of these responses was confirmed in the same study using the AHR antagonist CH223191 and the partial VDR antagonist 25(OH)D [12]. A remaining limitation is that target gene expression was measured only at the mRNA level; UVB- and FICZ-induced CYP1A1 protein expression in HaCaT keratinocytes has been independently confirmed by Western blot in an AHR-dependent manner [69]. However, but comparable protein-level validation of CYP24A1 in the HaCaT versus SCL-1 model remains unaddressed, leaving the functional relevance of the differential transcriptional responses in photocarcinogenesis to be confirmed by future studies.

The net immune outcome of UVB-driven VDR and AHR co-activation in skin is ligand-specific. FICZ-driven AHR activation promotes Th17 differentiation and accelerates onset and severity of EAE in mice [53], whereas kynurenine-driven AHR activation generates Foxp3^+^ regulatory T cells in wild-type but not AHR-null murine naïve T cells in vitro, establishing strict AHR-dependence [41]. 1,25D–VDR signalling reinforces the tolerogenic arm by inducing Foxp3 via a vitamin D response element in the mouse *Foxp3* promoter and by suppressing Th17 IL-17A production through VDR-mediated sequestration of Runx1 [59]. The cutaneous outcome therefore reflects the balance between FICZ (pro-Th17) and kynurenine (pro-Treg) AHR signals, modulated by VDR.

Kynurenine catabolism links the two pathways. IDO1 and TDO catalyse the rate-limiting conversion of tryptophan to kynurenine, an endogenous AHR ligand [49,50], and AHR reciprocally drives IDO expression in murine dendritic cells, forming a self-reinforcing loop [51]. A 1,25D → IDO limb completing this cascade has been demonstrated in rodent DCs [54], but is contradicted in human DCs, where 1,25D suppresses IDO activity and expression [55,56]—making the cascade species-restricted rather than universal. Because UVB simultaneously generates FICZ and triggers vitamin D_3_ synthesis from 7-dehydrocholesterol, skin is the tissue in which both pathways are co-activated by a single environmental stimulus, with the net effect determined by ligand identity and species-specific receptor architecture.

### 3.3. p53 as a Shared Transcriptional Activator of AHR and VDR

An underappreciated layer of AHR-VDR cross-regulatory integration involves the tumor suppressor p53. The *VDR* gene contains a functional p53 response element in its first intron, making it a direct transcriptional target of p53 [70]. AHR is also coupled to p53, but the regulatory architecture is more complex—operating through a non-canonical mechanism whose directionality differs between human and murine cells, as discussed in detail below [71]. Despite these mechanistic differences, both axes couple genotoxic stress signaling to vitamin D and xenobiotic receptor activity.

#### 3.3.1. AHR and p53

The functional relationship between AHR and p53 operates at two distinct molecular levels in human cells: post-transcriptional stabilisation of AHR protein [71] and direct p53-mediated transactivation of AHR target genes [72].

The first mechanism involves the p53-mediated stabilisation of AHR protein in response to genotoxic stress. The ataxia–telangiectasia mutated (ATM) protein kinase is activated by DNA double-strand breaks induced by agents such as doxorubicin and bleomycin, and in turn phosphorylates and activates p53 [73,74]. In normal human lung fibroblasts (WI-38) and osteosarcoma cells (Saos-2), engagement of this ATM–p53 axis by genotoxic stress increases AHR protein levels through a post-transcriptional mechanism, occurring without corresponding changes in *AHR* mRNA—confirmed by actinomycin D experiments showing that transcription inhibition does not block AHR protein upregulation [71]. Fibroblasts from ataxia–telangiectasia patients, which carry defective ATM signalling and impaired p53 activation, exhibit reduced baseline AHR protein levels and fail to upregulate AHR protein upon bleomycin treatment, providing direct genetic confirmation that intact ATM–p53 signalling is required for this stabilisation. The effect is also species-specific: in murine macrophage cell lines (RAW264.7 and J774.A1), p53 activation produces the opposite outcome, reducing both *Ahr* mRNA and AHR protein levels—a divergence that warrants caution when extrapolating findings between rodent models and human physiology [71].

The molecular route by which ATM–p53 signalling stabilises AHR protein remains incompletely defined. The cytoplasmic Hsp90/XAP2/p23 chaperone complex that determines baseline AHR stability is described in Section 2.2; whether p53 acts by modulating translation, altering p23–AHR binding, or shifting ubiquitination pathways remains to be determined by direct half-life measurements.

The second mechanism involves direct p53-mediated transactivation of AHR target genes rather than regulation of AHR itself. In an isogenic panel of HCT116 colorectal cells with defined *TP53* genotypes (wild-type, knockout, and a heterozygous R248W gain-of-function knock-in), benzo[a]pyrene-induced CYP1A1 protein expression was strongly p53-dependent: *TP53*-wild-type cells exhibited markedly higher CYP1A1 protein induction and significantly greater BaP-DNA adduct formation than p53-deficient or p53-mutant cells [72]. Bypassing the need for metabolic activation by treating cells directly with reactive PAH–diol-epoxide metabolites (BPDE, DB[a,h]ADE, DB[a,l]PDE) produced equivalent adduct levels across all genotypes, confirming that the influence of p53 operates at the level of PAH bioactivation rather than DNA repair. Mechanistically, ChIP analysis demonstrated that p53 binds a p53 response element in the 5′ region of the *CYP1A1* promoter, directly enhancing *CYP1A1* transcription. Importantly, AHR protein levels were not differentially regulated by p53 status; AHR decreased equally across all genotypes after PAH exposure, indicating that the regulation operates through *CYP1A1* transactivation rather than through changes in AHR expression [72].

Taken together, these findings indicate that wild-type p53 functions as a positive regulator of both AHR protein abundance and AHR-dependent xenobiotic gene transcription in human cells—a coherent model in which the receptor is stabilised post-transcriptionally and its target genes are simultaneously transactivated by p53 acting at their promoters. This produces a paradoxical implication for chemical carcinogenesis: although p53 functions canonically as a tumour suppressor, its enhancement of CYP1A1 activity may increase the bioactivation of polycyclic aromatic hydrocarbons into mutagenic metabolites in p53-competent cells, potentially explaining why *TP53*-wild-type cells form higher BaP-DNA adduct levels than p53-deficient cells [72]. This stands in contrast to the bidirectional architectural cross-talk observed between p53 and VDR—where the two transcription factors share co-localised binding sites on the *CDKN1A* and *MDM2* promoters—as no comparable evidence exists for direct co-localisation of p53 and AHR at the *AHR* locus itself; their convergence instead occurs at downstream target genes such as *CYP1A1*.

#### 3.3.2. VDR and p53

The p53 family comprises three structurally and functionally related transcription factors—p53, p63, and p73—that share a highly conserved DNA-binding domain with approximately 60% sequence homology and recognise closely related response elements [75,76]. While p53 is the most extensively characterised tumour suppressor and the most frequently mutated gene in human cancer, p63 and p73 contribute distinct yet overlapping roles in development, DNA damage responses, and tumour suppression [76]. All three members can exist as full-length transactivation-competent isoforms, allowing fine-tuned regulation of shared target genes [75,76].

The vitamin D receptor and the p53 family operate as mutually reinforcing tumour suppressors, with the dominant directional regulation flowing from p53 (and its family members) to VDR. A p53RE within the first intron of the *VDR* gene, consisting of two decameric half-sites of consensus RRRCWWGYYY, anchors this regulation: under conditions of forced p53 expression following genotoxic stress, p53 binds this element to directly induce *VDR* transcription and increase VDR protein expression, sensitising the cell to the anti-proliferative and pro-apoptotic effects of 1,25D [70].

The DNA damage response also induces VDR through this same regulatory architecture, but in this physiological context the principal inducer has been shown to be p73 rather than p53. Treatment of p53-null H1299 lung carcinoma and Saos-2 osteosarcoma cell lines with doxorubicin or etoposide increased p73 and VDR transcripts and protein levels, along with concomitant induction of *CYP27B1* mRNA; siRNA-mediated knockdown of p73 abolished DNA damage-induced VDR expression. p63γ also induced *VDR* transcription when overexpressed, whereas p53 itself did not induce VDR mRNA or protein at any dose tested [77].

The cross-talk becomes bidirectional at the level of shared target genes, with VDR- and p53-binding sites in close architectural proximity on multiple shared promoters. In MCF-7 breast cancer cells, ChIP analysis of the *CDKN1A* (p21^Waf1/Cip1^) promoter identified three VDR-binding regions, two of which also accommodate p53 binding [78]. A similar architectural arrangement has been described in osteoblasts at the *MDM2* P2 promoter—MDM2 being the principal negative regulator of p53—where a DR3-type VDR response element sits directly upstream of two p53 response elements within the same promoter region [79]. VDR also regulates additional p53 target genes, indicating functional overlap of the two networks [70,76]; these shared targets represent points of convergence through which VDR and the p53 family cooperate to control cell cycle arrest, differentiation, and apoptosis.

Critically, this cross-talk is inverted in cancers harbouring mutant p53, where gain-of-function mutant p53 binds VDR, promotes its nuclear accumulation, and converts 1,25D into an anti-apoptotic agent [80], meaning the tumour-suppressive function of the VDR–p53 axis depends on wild-type p53 status. The core mechanisms of AHR–VDR cross-regulation, together with their supporting evidence and current level of validation, are summarised in Table 1.

### 3.4. Genomic Co-Occupancy and Shared Transcriptional Targets

Genome-wide chromatin immunoprecipitation studies have separately characterised VDR and AHR/ARNT binding profiles—VDR in lymphoblastoid cells [85] and across multiple cell types including LPS-polarised THP-1 monocytes [86], and AHR/ARNT and AHR/AHRR in MCF-7 breast cancer cells [87,88]. However, no integrative cross-receptor genome-wide analysis directly comparing VDR–RXR and AHR–ARNT co-occupancy in matched cell types has yet been published. Available evidence for genomic cross-talk between the two receptor systems therefore derives from focused promoter-level studies of individual shared target genes.

The most extensively characterised shared target is the *CYP1A1* gene, which encodes the principal cytochrome P450 enzyme responsible for polycyclic aromatic hydrocarbon bioactivation. In human monocyte- and macrophage-derived cells (U937, THP-1) and in breast and kidney epithelial cells (MCF-7, HEK293), 1,25(OH)_2_D_3_ alone does not induce *CYP1A1*, but it markedly enhances benzo[a]pyrene (B[a]P)-induced-induced *CYP1A1* transcription, CYP1A1 protein expression, B[a]P hydroxylation activity, and B[a]P–DNA adduct formation [15]. This synergy is mediated by direct VDR–RXR binding to an everted-repeat type 8 (ER8) motif at positions −506 to −525 of the human *CYP1A1* promoter, located immediately adjacent to the AHR-responsive xenobiotic response element (XRE) at −489 to −495. This cis-cooperative architecture was demonstrated principally in U937 cells by electrophoretic mobility shift assay with mutant-competition and antibody-supershift, and confirmed by ChIP in BaP/1,25(OH)_2_D_3_-treated cells. Knockdown of *VDR* by siRNA, treatment with VDR antagonists, or treatment with the AHR antagonist α-naphthoflavone each abolishes the synergistic effect, indicating that both receptors are simultaneously required for full *CYP1A1* induction at this locus [15].

The cross-talk is bidirectional: at the *CYP24A1* gene encoding the principal vitamin D-inactivating enzyme, AHR activation by benzo[a]pyrene reciprocally enhances 1,25(OH)_2_D_3_-driven *CYP24A1* induction in human THP-1 monocyte/macrophage-derived cells and MCF-7 breast cancer cells, accelerating 1,25(OH)_2_D_3_ catabolism through CYP24A1-mediated 23- and 24-hydroxylation; the AHR antagonist α-naphthoflavone abolishes this enhancement, and de novo protein synthesis is required [13]. At both shared loci, therefore, VDR and AHR act as cooperative co-activators rather than antagonists, with one receptor’s ligand enhancing the other receptor’s transcriptional output.

The transcriptional outcome of this cross-talk shifts according to cellular state. In HaCaT keratinocytes representing the precancerous stage, UVB induces *CYP1A1* far more strongly than 1,25(OH)_2_D_3_, whereas in malignant SCL-1 keratinocytes the pattern is reversed—1,25(OH)_2_D_3_ becomes the stronger *CYP1A1* inducer. Notably, combined UVB and 1,25(OH)_2_D_3_ treatment markedly amplifies *CYP24A1* expression exclusively in malignant SCL-1 cells; this amplification was absent in HaCaT cells, indicating that the cooperative behaviour of the two receptors is reshaped during malignant progression [12]. The context-specific outcomes of these interactions across tissue settings are summarised in Table 2.

## 4. Immune Modulation: Convergence on Treg/Th17 Balance

### 4.1. AHR Signaling in T Cell Differentiation

AHR activation by the metabolically stable ligand TCDD produces sustained receptor signalling that drives Foxp3^+^ Treg differentiation, whereas the rapidly metabolised endogenous ligand FICZ produces transient activation that preferentially promotes Th17 differentiation [52]. Although originally interpreted as a ligand-intrinsic difference, this dichotomy is now understood to reflect the extent and duration of AHR signalling: when rapidly metabolised ligands are dosed to achieve TCDD-equivalent, sustained *Cyp1a1* induction, they suppress effector responses and expand Foxp3^+^ natural Tregs (alongside Foxp3^−^ Tr1 cells), whereas low, transient AHR activation instead enhances IL-17 production [81]. This underscores the role of ligand turnover in setting the Treg/Th17 balance. AHR-deficient mice show severely compromised Th17 effector function—IL-22 production is abolished and IL-17A markedly reduced—yet Th17 lineage commitment still occurs, indicating that AHR functions as an essential co-factor for the Th17 cytokine programme rather than as a lineage-defining factor [53,100]. The lineage-defining role is played by RORγt [101].

Human-cell evidence for AHR-driven Foxp3^+^ Treg induction is conditional. In human naive CD4^+^ T cells, TCDD-mediated AHR activation alone drives a Foxp3^−^ Tr1-like population rather than Foxp3^+^ Tregs; suppressive Foxp3^+^ iTreg cells are only obtained with TGF-β1 co-stimulation [60]. This co-requirement is species-relevant, since TGF-β1 alone induces suppressive Foxp3^+^ Tregs in mouse but not human naive CD4^+^ T cells [102]. The AHR–Treg arm therefore holds in human cells, but its output depends on the cytokine context.

### 4.2. VDR Signaling in T Cell Differentiation

The immunomodulatory actions of 1,25D on adaptive immunity are well established, with consistent replication across human and rodent systems and across multiple disease contexts [103,104]. In CD4^+^ T cells, 1,25D exerts a bidirectional effect on lineage commitment, suppressing pro-inflammatory Th1 and Th17 differentiation while favouring FOXP3^+^ regulatory T cell (Treg) generation. For Th1 cells, VDR/RXR directly represses *IFNG* via negative response elements in its promoter [105] and inhibits *IL2* by blocking NFATp/AP-1 complex formation at the NFAT-1 element [106]. For Th17 cells, 1,25D represses IL-17A through two complementary mechanisms: at the transcriptional level, VDR binds the proximal *IL17A*/*Il17a* promoter, competes with NFAT, sequesters Runx1, and recruits HDAC2, demonstrated in both human (Jurkat, HUT102) and mouse (EL-4) T cells [59], and post-transcriptionally, 1,25D suppresses Th17 cytokine output via CHOP induction, without altering *Rorc*, *Il17a*, or *Il23r* mRNA at physiological concentrations (0.1–10 nM) [107]. Concurrently, 1,25D enhances *FOXP3* transcription through VDR/RXR binding to three VDREs within the intronic conserved non-coding sequence at +1714 to +2554, a Treg-lineage enhancer [108]. Consistent with this, 1,25D induces CD4^+^FoxP3^+^ regulatory T cells with suppressive activity in vitro, acting both directly on human T cells in an IL-2-dependent manner [109] and indirectly through tolerogenic dendritic cell programming [110].

### 4.3. AHR–VDR Convergence on Tolerogenic Immune Programming

In vitro studies have shown that 1,25D suppresses Th17 differentiation from naive CD4^+^ T cells under standard polarising conditions, reducing IL-17A production through direct VDR-mediated repression of the *IL17A* locus [59] and lowering IL-17F output via CHOP-dependent post-transcriptional suppression—the latter reflecting reduced cytokine protein rather than transcriptional change, with *Rorc*/RORγt and Th17-cytokine mRNA unchanged at physiological concentrations (0.1–10 nM) [107]. 1,25D can concurrently favour FoxP3^+^ Treg induction via direct *Foxp3* transactivation [59]; however, this Treg-promoting effect is IL-2-dependent and is not seen under all conditions—where 1,25D lowers IL-2 availability it can suppress Treg as well as Th17 differentiation [89]. The Treg-skewing effect is more robust when 1,25D acts via dendritic cell priming rather than directly on T cells [56]. Whether 1,25D specifically antagonises AHR-driven (FICZ-induced) Th17 polarisation, or operates through parallel Th17-suppressive pathways—such as direct VDR binding to the *IL17A* locus [59]and miR-124-mediated downregulation of IL-6R, which lowers downstream STAT3 phosphorylation [90]—has not been directly tested in head-to-head experiments.

**Figure 1:**
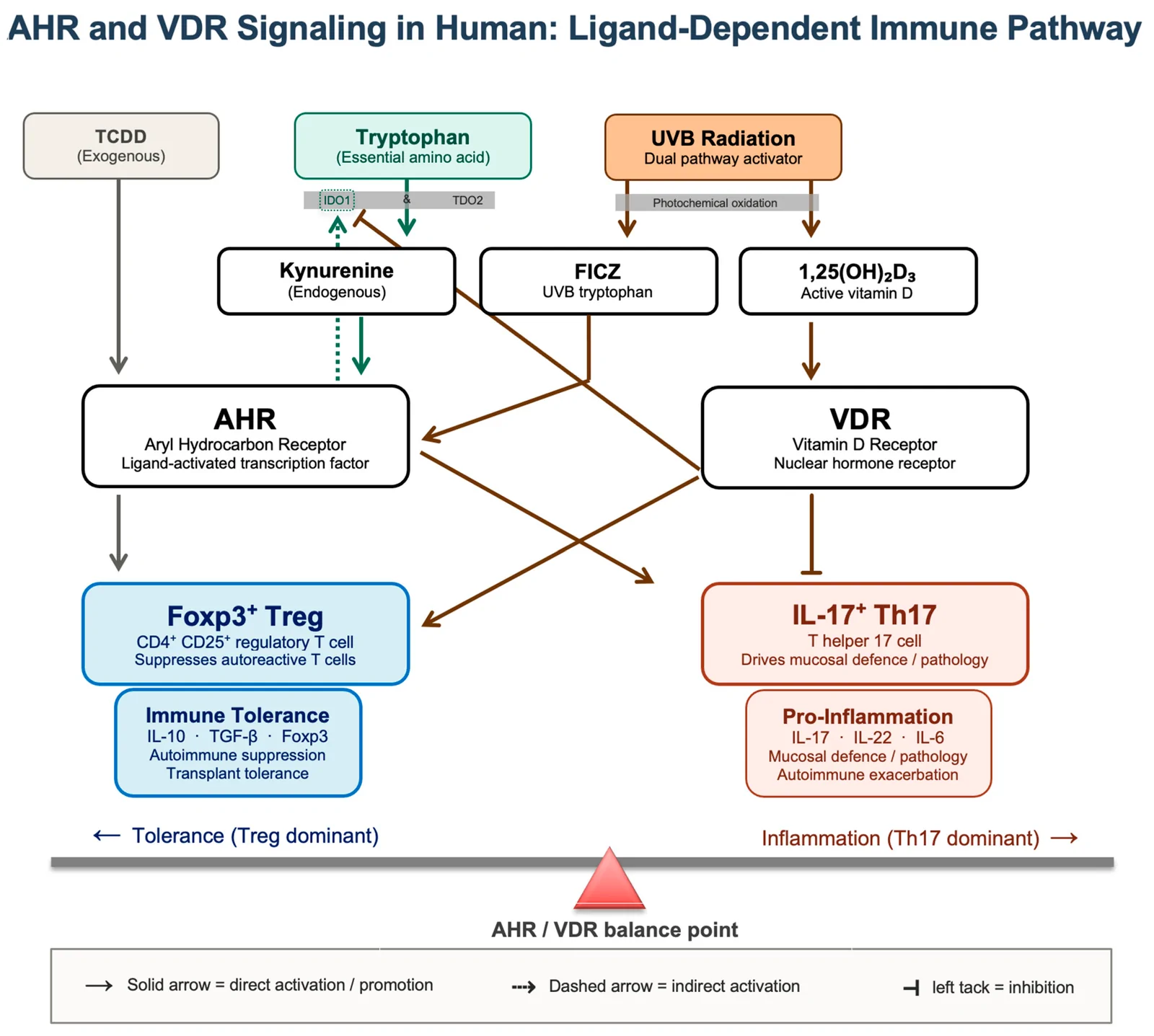
**Ligand-dependent AHR and VDR signaling**. TCDD drives AHR activation toward Foxp3^+^ Treg induction and immune tolerance. FICZ drives transient AHR activation toward IL-17^+^ Th17 promotion and inflammation. VDR suppresses IDO1 activity, independently promotes Foxp3^+^ Treg differentiation, and inhibits Th17 differentiation. AHR = aryl hydrocarbon receptor; VDR = vitamin D receptor; FICZ = 6-formylindolo[3,2-b]carbazole; TCDD = 2,3,7,8-tetrachlorodibenzo-p-dioxin; IDO1 = indoleamine 2,3-dioxygenase 1; TDO2 = tryptophan 2,3-dioxygenase.

A second, more indirect layer of cross-regulation has been proposed at the level of the tryptophan metabolite environment that supplies AHR ligands. 1,25D promotes the differentiation of IDO-expressing tolerogenic dendritic cells that enhance FoxP3^+^ regulatory T cell generation, as shown in a rat model of autoimmune encephalomyelitis [54]; more broadly, 1,25D-induced tolerogenic dendritic cells drive CD4^+^CD25^+^ regulatory T cells in mouse models of autoimmunity, albeit without a defined IDO requirement [111]. In TLR-stimulated murine dendritic cells, AHR is itself required upstream of IDO induction, and the resulting kynurenine supports tolerogenic IL-10 secretion and Treg promotion [51]. Tryptophan-derived endogenous AHR ligands converge on this Treg-promoting axis through distinct routes: kynurenine, the first metabolite of the IDO pathway, engages AHR directly within T cells to drive FoxP3^+^ Treg differentiation [41], whereas the indole–thiazole ligand ITE induces FoxP3^+^ Tregs both by acting on T cells and by programming tolerogenic dendritic cells, suppressing EAE in vivo [112]. The contribution of this IDO–AHR axis to vitamin D3-driven tolerogenicity in human cells, however, remains less well characterised. IDO-independent mechanisms also operate in human dendritic cells exposed to vitamin D3 in combination with IFN-γ: the pharmacological IDO inhibitor epacadostat fails to abrogate the tolerogenic phenotype, which depends instead on MEK/ERK and PI3K/mTOR signalling [113]. The assumption that kynurenine itself is the operative AHR ligand has also been challenged: in vitro kynurenine preparations may activate AHR primarily through trace condensation derivatives rather than through the parent molecule, with kynurenine acting as a pro-ligand [114]. Furthermore, kynurenine is generated enzymatically from tryptophan by IDO1/TDO, whereas FICZ is a UV photoproduct of tryptophan; the two metabolites arise through distinct pathways. No published demonstration exists that 1,25D treatment measurably depletes endogenous FICZ, although indirect effects via modulation of CYP1A1-mediated FICZ clearance cannot be ruled out and have not been systematically tested. It therefore remains incompletely defined whether metabolite reconfiguration or direct receptor-level cross-talk drives the net AHR–VDR antagonism in Th17/Treg balance. An overview of ligand-dependent AHR and VDR signaling is presented in Figure 1.

## 5. Intestinal Epithelium, Gut Microbiota, and Inflammatory Bowel Disease

### 5.1. AHR in the Intestine and the Microbiome Connection

AHR signalling is functional in intestinal epithelial cells, in lamina propria immune cells [115,116], in group 3 innate lymphoid cells (ILC3) [117,118], in intraepithelial lymphocytes [119], in Th17 cells [100], and—as more recently established—in enteric neurons [120]. *Lactobacillus reuteri* produces IAld via an ArAT-dependent indole-3-pyruvate pathway in the gut, activating AHR and driving IL-22 production by ILC3s; *Lactobacillus acidophilus* mediates the same IAld–AHR–IL-22 axis in the vaginal mucosa in an organ-specific manner [91]. Tryptophanase-competent commensals generate indole from tryptophan, while separate bacterial pathways yield indole-3-acetic acid (IAA); both indole and IAA are confirmed AHR ligands [92].

*Peptostreptococcus russellii* produces indoleacrylic acid (IA) via a reductive phenyllactate dehydratase (fldAIBC) pathway; IA activates AHR (inducing *Cyp1a1*) in murine intestinal epithelial cells and additionally engages the NRF2-ARE antioxidant pathway in immune cells [121]. A homologous fldAIBC reductive pathway in *Clostridium sporogenes*—and in *Peptostreptococcus anaerobius* and *Clostridium cadaveris*, which carry related gene clusters—instead reduces the intermediate fully to indolepropionic acid (IPA) [122]; IPA fortifies the epithelial barrier via the pregnane X receptor (PXR) rather than AHR [123]. The physiological importance of this microbial indole axis is underscored by genetic evidence in both mice and humans. Caspase recruitment domain family member 9 (CARD9) deficiency reduces the abundance of tryptophan-catabolising bacteria including *L. reuteri*, impairing AHR ligand production and AHR-dependent IL-22 responses. A homologous impairment has been observed in IBD patients carrying the CARD9 rs10781499 risk allele [124]. Multiple ligand sources and receptor pathways thus converge to regulate intestinal barrier defence; as the diversity of characterised pathways suggests, no single microbial metabolite is likely to be the dominant mediator under all physiological conditions [92].

### 5.2. VDR in the Intestine

The intestine has one of the highest levels of VDR expression of any tissue [125]. VDR signalling supports intestinal epithelial barrier integrity by directly upregulating the tight junction protein (TJP) ZO-1 and maintaining occludin localisation at the tight junction complex [93], and by transcriptionally activating claudin-15—a channel-forming claudin whose loss increases epithelial permeability and whose expression is reduced in inflammatory bowel disease, particularly ulcerative colitis [94]. VDR also directly transactivates *CLDN2* [126]; claudin-2 is, however, a pore-forming “leak” claudin elevated in active IBD, so this represents a distinct, context-dependent arm of VDR’s barrier regulation rather than a barrier-tightening effect. At the level of inflammatory signalling, VDR restrains bacterially stimulated NF-κB activation in intestinal epithelial cells—physically interacting with the p65 subunit to limit its nuclear translocation—thereby suppressing pro-inflammatory signalling and protecting against enteric bacterial challenge [95]. Murine models support a protective role: whole-body *Vdr*-knockout mice develop more severe colitis in CD4^+^CD45RB^hi^ T-cell-transfer and IL-10-knockout models [127]; epithelial VDR overexpression protects against TNBS-, DSS-, and transfer-induced colitis [128]; and conversely, intestinal-epithelial-specific *Vdr* deletion (VDRΔIEC) increases colitis susceptibility [94]. Polymorphisms in the VDR gene (TaqI, FokI, ApaI, BsmI) have been associated with susceptibility to Crohn’s disease and ulcerative colitis in several populations, although findings are inconsistent across cohorts [129,130].

### 5.3. AHR-VDR Cooperation in Intestinal Homeostasis

Both pathways suppress NF-κB activation in the intestinal epithelium—VDR by physically interacting with the p65 subunit and limiting its nuclear translocation, such that VDR deficiency elevates epithelial NF-κB activity and worsens colitis [95,128]. AHR contributes in parallel by suppressing cytokine-induced NF-κB p65 signalling [131]. Both also help preserve epithelial tight-junction organization under barrier-disrupting challenge—VDR by maintaining ZO-1 expression and stabilizing occludin localization [93], and AHR by protecting the membrane localization of ZO-1, occludin, and tricellulin [132]. Both pathways are also associated with regulatory T cell responses that protect against intestinal inflammation, albeit through distinct mechanisms. Vitamin D sufficiency increases the frequency of colonic FoxP3+ and RORγt+FoxP3+ Tregs and reduces colitis susceptibility in mice, an effect mediated indirectly through vitamin D-dependent shaping of the microbiota [133]. AHR activation expands FoxP3+ Tregs in the intestinal lamina propria and restores the colitis-associated Treg/Th17 imbalance, in part by reversing aberrant methylation of the Foxp3 promoter [134]. Taken together, these parallel actions suggest that AHR and VDR signalling converge on protective, anti-inflammatory functions in the intestinal epithelium and mucosal immune compartment under conditions of inflammatory or metabolic challenge, even though direct evidence for integrated cross-talk between the two pathways in this tissue remains limited.

In IBD, both pathways are frequently compromised in parallel. Vitamin D deficiency is significantly more prevalent among IBD patients than in controls [96] and, in Crohn’s disease specifically, is independently associated with greater disease activity and lower health-related quality of life [135]. Independently, IBD-associated dysbiosis is characterised by reduced abundance of tryptophan-metabolising bacteria and decreased fecal concentrations of AHR-activating indole derivatives such as indole-3-acetic acid [124]; *AHR* expression itself is reduced in inflamed intestinal tissue from IBD patients compared with healthy controls [115]. The convergence of impaired VDR signalling (via deficiency or VDR downregulation in inflamed mucosa) and impaired AHR signalling (via reduced ligand supply and reduced receptor expression) may therefore represent a compounded deficit in anti-inflammatory signalling. Whether the two impairments interact mechanistically in IBD pathogenesis, however, remains to be directly tested.

## 6. Skin Biology and Photobiology

### 6.1. Keratinocytes as a Dual-Receptor System

The skin is unique in that solar UVB radiation simultaneously initiates both VDR and AHR signalling through distinct photochemical routes. UVB converts epidermal 7-dehydrocholesterol to vitamin D_3_, which keratinocytes further metabolise to the active hormone 1,25(OH)_2_D via CYP27A1 and CYP27B1 [68,136]. In parallel, UVB generates an AHR-activating tryptophan photoproduct, proposed to be the high-affinity agonist 6-formylindolo[3,2-b]carbazole (FICZ). This rapidly induces AHR nuclear translocation, site-specific DNA binding, and target gene regulation in keratinocytes via circulating photoproducts in peripheral tissues, including the intestine (demonstrated in mice) [137]. Keratinocytes express both VDR and AHR, positioning them as a convergence point for these two UV-driven pathways [12,136]. Through VDR, 1,25(OH)_2_D inhibits keratinocyte proliferation and promotes sequential epidermal differentiation [68,136]. Notably, the two pathways share downstream target genes in keratinocytes: 1,25(OH)_2_D induces the canonical AHR target CYP1A1 in an AHR-dependent manner, as demonstrated by abrogation with the AHR antagonist CH223191. Combined UVB and 1,25(OH)_2_D treatment synergistically upregulates the VDR target CYP24A1 in malignant keratinocytes [12]. These observations point to bidirectional cross-talk between VDR and AHR signalling in this cell type, though the molecular mechanism linking the two pathways—and whether it operates under physiological as opposed to pharmacological or carcinogenic conditions—remains to be defined.

### 6.2. Implications for Psoriasis and Atopic Dermatitis

In psoriasis, both 1,25D analogs (calcipotriol) and AHR modulators have demonstrated therapeutic efficacy. Tapinarof, a bacterially derived AHR agonist [138], is approved by the FDA for plaque psoriasis in adults [139] on the basis of phase 3 trial efficacy [140]. Tapinarof activates AHR in primary human keratinocytes and reduces Th17-driven IL-17A responses in ex vivo human skin and CD4+ T cells; IL-17F suppression has been demonstrated in murine models [138]. The AHR-agonist clinical evidence base has since been extended to atopic dermatitis: in the pivotal Phase 3 ADORING 1 and ADORING 2 trials, tapinarof cream 1% once daily met the primary vIGA-AD endpoint at Week 8 with statistical significance in adults and children ≥2 years, corresponding to the December 2024 FDA approval extension for atopic dermatitis [141]. Tapinarof additionally upregulates epidermal differentiation markers including filaggrin, hornerin, and involucrin in primary human keratinocytes, supporting barrier restoration [138,140]. VDR analogs achieve overlapping effects through direct suppression of human keratinocyte [142,143] and through attenuation of the IL-23/IL-17 axis, demonstrated via keratinocytic VDR signaling in murine psoriasis models and corroborated in lesional skin from human plaque psoriasis patients [144]. Calcipotriol further suppresses Th17 cytokine-induced S100 alarmins (psoriasin and koebnerisin) in human keratinocytes [145]. Given their mechanistically distinct but convergent anti-inflammatory actions on keratinocytes, we propose that co-targeting VDR and AHR pathways with calcipotriol and tapinarof may represent a rational therapeutic strategy warranting formal clinical investigation.

## 7. Cancer Cell Biology

### 7.1. VDR as a Tumor Suppressor

Extensive epidemiological, genetic, and experimental evidence supports an anti-tumor role for VDR signaling. 1,25D, acting through VDR, transcriptionally induces the cyclin-dependent kinase inhibitor p21 via a functional VDRE in the p21 promoter, driving cell cycle arrest in cancer and leukemic cells [97], and concurrently upregulates p27 [7]. 1,25D also induces apoptosis through mitochondrial disruption involving BAX translocation and cytochrome c release, with downregulation of the anti-apoptotic protein Bcl-2 [7,146]. It also enhances expression of the epithelial differentiation marker E-cadherin [7]. In parallel, 1,25D antagonizes growth-promoting pathways, including Wnt/β-catenin and epidermal growth factor receptor signaling [7], and suppresses Hedgehog pathway components (Shh, Ptch1, Smoothened, Gli1, Gli2) in murine keratinocytes and epidermal explants [147]. The p53 family has also been linked to VDR regulation, although the relationship is more nuanced than a simple p53→VDR axis. A conserved p53 response element resides within the first intron of the *VDR* gene; ectopically expressed p53 binds this element and induces *VDR* transcription [70]. Functionally, VDR overexpression was required for vitamin D_3_-mediated induction of *CDKN1A* (p21) in HCT116 colon cancer cells, linking elevated VDR expression to 1,25D sensitivity [70]. This induction, however, was demonstrated under forced (adenoviral) p53 expression rather than physiological genotoxic stress; following DNA damage, p53 itself does not induce VDR, with the related family member p73 serving as the principal inducer and p63 also regulating VDR [77]. Independently, VDR expression is lost in advanced and high-grade colon tumors through SNAIL-mediated transcriptional and post-transcriptional repression of the *VDR* promoter, an event linked to epithelial-to-mesenchymal transition that causes unresponsiveness to 1,25D and its analogs [98,99].

### 7.2. AHR in Cancer: Context-Dependent Roles

The role of AHR in cancer biology is complex and context-dependent. Activation of AHR by polycyclic aromatic hydrocarbons such as benzo[a]pyrene induces *CYP1A1*, generating reactive intermediates that form DNA adducts and mediate carcinogenesis; this carcinogenicity is abolished in AHR-null mice [148]. In other contexts, AHR functions as a tumor suppressor: AHR-null mice spontaneously develop cecal tumors, and dietary natural ligands such as indole-3-carbinol and 3,3′-diindolylmethane suppress intestinal tumorigenesis in *Apc*^Min/+^ mice through AHR-dependent β-catenin degradation [149]. Conversely, constitutively active AHR—sustained by tryptophan-derived endogenous ligands generated through a kynurenine pathway amplification loop—enforces cell migration in triple-negative breast cancer cells [150]. AHR also exhibits functional crosstalk with the p53 stress response. DNA-damaging agents that activate p53 post-transcriptionally elevate AHR protein levels in human cells, with concurrent induction of *AHR* target genes, while producing the opposite effect in murine cells [71]. Activated AHR in turn transcriptionally induces the E2 ubiquitin-conjugating enzyme Ube2l3 via xenobiotic-response elements in the *Ube2l3* promoter, which together with the E3 ligase E6AP promotes p53 ubiquitination and proteasomal degradation, attenuating apoptosis [82]. A similar *UBE2L3* induction is observed with the natural AHR ligand indole-3-carbinol in human cervical cancer cells, where it is associated with decreased proliferation [83]. Genetic ablation of AHR in p53-deficient mice shortens lifespan and accelerates tumorigenesis compared to p53 loss alone, indicating that AHR cooperates with p53 as a tumor suppressor [84].

### 7.3. VDR–AHR–p53 Triple Interaction in Cancer Cells

The three-way interaction among VDR, AHR, and p53 in cancer cells represents a critical regulatory hub in which the DNA damage response, xenobiotic surveillance, and vitamin D signaling converge. p53 has been identified as a direct transcriptional regulator of VDR, binding a p53RE in the first intron of the *VDR* gene to induce *VDR* transcription when ectopically expressed, thereby sensitizing cancer cells to the anti-proliferative effects of 1,25D [70]. This induction, however, was demonstrated under forced p53 expression; following genotoxic stress p53 itself does not induce VDR—the related family member p73 is the principal inducer, with p63 also regulating VDR [77]. DNA-damaging agents also elevate AHR protein levels post-transcriptionally in human cells, with concurrent induction of AHR target genes, indicating that p53 activation couples both receptor systems to the genotoxic stress response [71].

A central node of this convergence is the cyclin-dependent kinase inhibitor p21Cip1/Waf1 (*CDKN1A*). The human p21 promoter contains multiple functional VDREs and p53REs, mapped by ChIP and reporter assays in MCF-7 breast cancer cells [78]. AHR also engages this promoter: acting as a heterodimeric complex with the tumor suppressor Krüppel-like factor 6, it binds a non-consensus xenobiotic response element (NC-XRE) on the p21 promoter to drive p21Cip transcription, as demonstrated in mouse hepatocytes during liver regeneration [151]. AHR-dependent p21 induction has been further confirmed in mouse Hepa1 and human HepG2 hepatoma cells, where the AHR ligand SU5416 induced p21^cip1/waf1^ and G1 arrest in a manner requiring both AHR and ARNT [152]. The cooperative nature of p21 regulation is illustrated by the dependence of vitamin D-mediated growth inhibition on tumor suppressor co-factors: in MCF-7 breast cancer cells, vitamin D analogs require BRCA1 to recruit VDR to three VDRE sites in the *CDKN1A* promoter, enhance histone acetylation, and induce p21^waf1^. Knockdown of either BRCA1 or p21^waf1^ abolishes growth inhibition [153]. The convergence of VDR, AHR, and p53 on p21 therefore positions this gene as an integrative checkpoint where multiple anti-tumor pathways are coordinated.

The integrated axis also includes a regulatory brake: activated AHR transcriptionally induces the E2 ubiquitin-conjugating enzyme *Ube2l3* via xenobiotic-response elements in its promoter, which together with the E3 ligase E6AP promotes p53 ubiquitination and proteasomal degradation, thereby attenuating excessive p53-driven apoptosis [82]. This feedback mechanism may limit p53-mediated cell death during DNA repair, although the integration of this brake with the cooperative p21-inductive arm in cancer cells remains to be defined.

Mutant p53 (mutp53) physically and functionally interacts with VDR, is recruited to VDR-regulated genes, increases nuclear VDR accumulation, and importantly converts 1,25D from a pro-apoptotic to an anti-apoptotic agent—demonstrated in mutp53-expressing breast cancer cells (SKBR3, MDA-MB-231) and in p53-null H1299 lung adenocarcinoma cells engineered to express mutp53 [80]. In contrast, in gastric cancer cells (TMK1), mutp53 is essential for 1,25D-induced p21 upregulation and growth arrest, indicating that mutant p53 stabilizes VDR through direct interaction and mediates rather than abolishes vitamin D-induced anti-tumor effects [154]. The influence of p53 status on VDR output is further illustrated in keratinocytes. 1,25D induced the VDR target *CYP24A1* exclusively in p53-null SCL-1 cells and not in p53-mutant HaCaT cells, indicating that p53 status (wild-type, mutant, or null) qualitatively alters the output of the integrated VDR–AHR–p53 response, though in this instance in the direction of greater VDR-target induction in the p53-null line [12].

A further pharmacologically relevant consequence of AHR–VDR cross-talk is the AHR-driven induction of *CYP24A1*, the principal vitamin D-inactivating enzyme. In human THP-1 monocytic and MCF-7 breast cancer cells, the AHR ligand benzo[a]pyrene enhanced 1,25D-induced *CYP24A1* expression and accelerated 23-/24-hydroxylation of 1,25D, demonstrating that AHR activation can blunt VDR-mediated growth suppression through accelerated catabolism of the active hormone [13]. Reciprocally, 1,25D enhances BaP-induced *CYP1A1* expression in human U937, THP-1, and breast and kidney cell lines through a functional everted repeat (ER8) motif in the *CYP1A1* promoter, identifying *CYP1A1* as a novel VDR target gene and revealing bidirectional cross-talk that may modulate both xenobiotic activation and detoxification in tumor cells [15].

## 8. Conclusions

The aryl hydrocarbon receptor and the vitamin D receptor, long studied as the separate controllers of xenobiotic defence and calcium–vitamin D homeostasis, emerge from the evidence assembled here as two arms of a single, integrated regulatory system. Both are ligand-activated transcription factors that respond to their chemical environment—one to planar aromatic and tryptophan-derived signals, the other to a secosteroid hormone—and both drive overlapping transcriptional programmes. Rather than operating in isolation, they are linked at multiple levels, and the recognition of this cross-talk reframes each receptor as a node within a shared network rather than a self-contained pathway.

The mechanistic basis of this integration is now reasonably well defined. The two receptors draw on a common pool of coactivators (SRC/p160, CBP/p300) and cooperate at composite promoters. This is most clearly seen where an everted repeat (ER8) vitamin D response element sits immediately adjacent to a canonical XRE in the *CYP1A1* promoter, allowing VDR–RXR and AHR–ARNT to act together rather than in competition. The relationship is reciprocal: AHR activation by benzo[a]pyrene or TCDD enhances 1,25D-dependent induction of *CYP24A1* and accelerates vitamin D catabolism. Tryptophan metabolism supplies a further bidirectional link, with kynurenine and the UVB photoproduct FICZ serving as endogenous AHR ligands. Overlying these is the tumour suppressor p53, which couples genotoxic stress to both receptors—inducing *VDR* through a response element in its first intron while stabilising AHR protein post-transcriptionally—and converges with both on the *CDKN1A* (p21^Cip1/Waf1^) checkpoint. These core mechanisms, together with their supporting evidence, are summarised in Table 1.

A recurring and defining theme is that the *direction* of this cross-talk is not fixed but contingent. Whether AHR and VDR reinforce or oppose one another depends on cell type, ligand identity and kinetics, promoter architecture, malignant state, and—critically—species. The tryptophan–IDO1 axis, which supports tolerogenic signalling in rodent dendritic cells but is reversed in their human counterparts, is the clearest cautionary example. The p53 axis is similarly conditional: mutant p53 converts 1,25D from a pro-apoptotic to an anti-apoptotic signal in breast and lung cancer cells, yet is required for 1,25D-induced growth arrest in gastric cancer cells. Table 2 sets out these context-specific outcomes side by side across the tissue and pathway settings covered in this review.

At the physiological level, this convergence favours restraint: promotion of Foxp3^+^ regulatory T cells over Th17 effectors, suppression of NF-κB and preservation of the intestinal barrier, coordinated responses to solar UVB in the skin, and cooperative enforcement of cell-cycle arrest in transformed cells. That the two pathways are frequently impaired together, as in inflammatory bowel disease, and can be targeted together, as proposed here for calcipotriol and tapinarof in psoriasis, underscores the clinical importance of understanding their interaction.

Important gaps nonetheless remain. Direct competition for shared coactivators has never been demonstrated for this specific receptor pair; no integrative genome-wide analysis has compared VDR–RXR and AHR–ARNT co-occupancy in matched cell types; whether the two gene loci achieve physical proximity in nuclear space is untested; and it remains unknown whether 1,25D directly antagonises FICZ-driven Th17 polarisation or acts through parallel pathways. Resolving these—with explicit attention to human validation and to physiological rather than pharmacological conditions—is the necessary next step. Positioned as convergent environmental sensors, AHR and VDR offer both a unifying framework for how environmental, nutritional, and genotoxic signals are integrated at the level of transcription, and a rational basis for therapies that engage the two pathways together across immunology, mucosal biology, dermatology, and oncology.

## Figures and Tables

**Table 1:** Key Mechanistic Insights into AHR–VDR Cross-Regulation.

Core Mechanisms of AHR–VDR Cross-Talk	References
1. CYP24A1 induction by AHR: AHR ligands (BaP, TCDD) enhance 1,25D-dependent *CYP24A1* induction in THP-1 and MCF-7 cells, accelerating 1,25D catabolism and reducing VDR-ligand bioavailability.	[13,43]
2. IDO1–tryptophan axis (species-divergent): In rat bone-marrow DCs, vitamin D3 induces IDO^+^ tolerogenic DCs that expand Foxp3^+^ Treg. In human monocyte-derived DCs, 1,25D instead down-modulates IDO activity, and the tolerogenic effect operates through IDO-independent mechanisms.	[54,55,56]
3. FICZ vs. TCDD ligand specificity: AHR directionality on the Th17/Treg axis reflects the extent and duration of signalling rather than ligand identity per se. Sustained activation (TCDD, or rapidly metabolised ligands dosed to TCDD-equivalent exposure) expands Foxp3^+^ Treg; low, transient activation (FICZ) enhances IL-17.	[52,81]
4. p53 as a shared upstream regulator: p53 directly induces *VDR* via a p53RE in its first intron, whereas AHR protein is raised post-transcriptionally rather than through a p53RE in the *AHR* locus. p53 and VDR share co-localised binding sites at *CDKN1A* and *MDM2*; no comparable p53–AHR co-localisation exists at the *AHR* locus, their convergence occurring instead at downstream targets such as *CYP1A1*.	[70,71,72]
5. VDR–p53 synergy: The p53RE in *VDR* intron 1 mediates genotoxic-stress-induced VDR upregulation, sensitising cells to the anti-proliferative effects of calcitriol. The wider p53 family (p53, p63, p73) shares a conserved DNA-binding domain and recognises closely related response elements.	[70,75,76]
6. AHR–p53 check-and-balance: During genotoxic stress the ATM–p53 axis increases AHR protein post-transcriptionally in human cells (opposite in murine cells). Activated AhR then transcriptionally induces *Ube2l3* via XREs, which with the E3 ligase E6AP promotes p53 ubiquitination and proteasomal degradation, attenuating apoptosis.	[71,82,83,84]
7. Chromosomal gene proximity: *AHR* (Chr 7, 7p21.1) and *VDR* (Chr 12, 12q13.11) lie on distinct chromosomes, confirming genetic independence at the linear DNA level. Whether the loci achieve physical proximity in 3D nuclear space remains to be demonstrated and is an important open question.	[10,14,15]
8. Cell-type specificity: In keratinocytes and monocyte/macrophage-derived cells (U937, THP-1) AHR and VDR act cooperatively; antagonism is documented in hepatocytes. Ligand identity, promoter architecture, and malignant state are key determinants.	[12,15,37]

**Table 2:** Key AHR-VDR Cross-Regulatory Interactions by Context.

Pathway/Context	VDR Effect	AHR Effect	Net Cross-Talk	References
Immune (Th17/Treg)	↑ FOXP3^+^ Treg; represses Th17—VDR binds the *IL17A* promoter, competes with NFAT, sequesters Runx1, recruits HDAC2	Direction set by signalling duration: sustained (TCDD, kynurenine) → Foxp3^+^ Treg; transient (FICZ) → Th17	Both converge on Treg induction as parallel pathways. Whether 1,25D directly antagonises FICZ-driven Th17 has not been tested head-to-head.	[52,59,81,89,90]
CYP1A1 transcription	Liganded VDR–RXR binds an everted-repeat (ER8) VDRE at −506 to −525 bp and enhances—does not repress—transcription	AHR–ARNT induction via the adjacent canonical XRE at −489 to −495 bp	Cooperative, synergistic activation at a composite promoter—not competition. Abolished by VDR knockdown, VDR antagonist, or AHR antagonist (α-naphthoflavone).	[15]
CYP24A1 (1,25D catabolism)	Canonical VDR target; strong 1,25D-dependent induction	AHR ligands (BaP, TCDD) enhance the 1,25D-DEPENDENT induction; requires de novo protein synthesis and is blocked by α-naphthoflavone (indirect—no AHR element mapped at this locus)	AHR activation accelerates 1,25D catabolism, reducing VDR-ligand availability	[13,43]
Gut barrier/IBD	↑ Tight junction (ZO-1, occludin, claudin-15); restrains NF-κB via p65 interaction	Microbial indoles → IL-22 → barrier defence and antimicrobial peptides	Parallel convergence on barrier protection; both pathways impaired together in IBD, though direct evidence for integrated cross-talk in this tissue remains limited.	[91,92,93,94,95,96]
Cancer (breast, colon)	Anti-proliferative, pro-apoptotic; tumour suppressor	Context-dependent—pro- or anti-tumorigenic	Co-activation enhances xenobiotic-metabolising enzyme expression, with implications for carcinogenic risk under combined PAH and vitamin D exposure	[7,13,97,98,99]
Skin/keratinocytes	Promotes differentiation; 1,25D induces *CYP1A1* and *CYP24A1*	UVB → FICZ → AHR activation	Direction depends on cell state: combined UVB + 1,25D amplifies *CYP24A1* ONLY in malignant SCL-1 cells and is absent in precancerous HaCaT cells.	[12]
Tryptophan metabolism	SPECIES-DIVERGENT: 1,25D induces IDO^+^ tolerogenic DCs in rat, but DOWN-modulates IDO activity in human monocyte-derived DCs	Kynurenine is an endogenous AHR ligand promoting Treg differentiation	The 1,25D → IDO1 → kynurenine → AhR bridge is largely rodent-restricted; in humans Treg induction proceeds by IDO-independent routes.	[41,54,55,56]
p53/cell stress	p53RE in *VDR* intron 1 → direct transcriptional induction; p53 and VDR share co-localised sites at *CDKN1A* and *MDM2*	p53 raises AHR protein post-transcriptionally (stabilisation, not transcription); AHR then induces *Ube2l3* → E6AP-mediated p53 degradation	Convergence at the p21/*CDKN1A* checkpoint; mutant p53 inverts the VDR axis in a tissue-dependent manner	[70,71,72,82]

↑ = increase/upregulation; → = leads to.

## Data Availability

No new data were created or analyzed in this study. Data sharing is not applicable to this article.

## Abbreviations

1,25D = 1,25-dihydroxyvitamin D3; AHR = aryl hydrocarbon receptor; ARNT = AHR nuclear translocator; VDR = vitamin D receptor; RXR = retinoid X receptor; XRE = xenobiotic response element; VDRE = vitamin D response element; p53RE = p53 response element; CYP = cytochrome P450; TCDD = 2,3,7,8-tetrachlorodibenzo-p-dioxin; FICZ = 6-formylindolo[3,2-b]carbazole; IDO1 = indoleamine 2,3-dioxygenase 1; Treg = regulatory T cell; Th17 = T helper 17 cell.
